# Modulation of lethal HPAIV H5N8 clade 2.3.4.4B infection in AIV pre-exposed mallards

**DOI:** 10.1080/22221751.2020.1713706

**Published:** 2020-01-23

**Authors:** Susanne Koethe, Lorenz Ulrich, Reiner Ulrich, Susanne Amler, Annika Graaf, Timm C. Harder, Christian Grund, Thomas C. Mettenleiter, Franz J. Conraths, Martin Beer, Anja Globig

**Affiliations:** a Friedrich-Loeffler-Institut, Greifswald, Germany; b Institute of Veterinary-Pathology, Leipzig University, Leipzig, Germany

**Keywords:** Seropositive, mallard duck, HPAIV H5N8, clade 2.3.4.4 B, reservoir host, AIV pre-exposure

## Abstract

In 2016/2017, a severe epidemic of HPAIV H5N8 clade 2.3.4.4 group B (H5N8B) affected Europe. To analyse the role of mallards in the spatiotemporal dynamics of global HPAIV H5N8B dispersal, mallards (*Anas platyrhynchos*), naturally exposed to various AIV and therefore seropositive, were challenged with H5N8B. All experiments were controlled by infection and co-housing of seronegative juvenile Pekin ducklings. All ducks that survived the first infection were re-challenged 21 dpi with the homologous H5N8B strain. After the first H5N8B infection, seropositive mallards showed only mild clinical symptoms. Moderate to low viral shedding, occurring particularly from the oropharynx and lasting for 7 days maximum, led to severe clinical disease of all contact ducklings. All challenged seronegative Pekin ducks and contact ducklings died or had to be euthanized. H5-specific antibodies were detected in surviving birds within 2 weeks. Virus and viral RNA could be isolated from several water samples until 6 and 9 dpi, respectively. Conversely, upon re-infection with homologous H5N8B neither inoculated nor contact ducklings showed any clinical symptoms, nor was an antibody titer increase of seropositive mallards or any seroconversion of contact ducklings observed. Mallard ducks naturally pre-exposed to LPAIV can play a role as a clinically unsuspicious virus reservoir for H5N8B effective in virus transmission. Mallards with homologous immunity did not contribute to virus transmission.

## Introduction

In 1996, a highly pathogenic avian influenza (HPAI) H5N1-lineage emerged from domestic poultry in China (Goose/Guangdong/96 – Gs/Gd/96) as an ancestral virus for subsequent circulation, adaptation and differentiation into reassortant derivates that widely dispersed over vast distances [1]. HPAIV H5N8 clade 2.3.4.4 first appeared around 2010 in China [2] and reached Europe in autumn 2014 (group A) [3,4]. A new and phylogenetically distinct group of the same clade (B) was then detected in October/November 2016 in Europe [5]. The epidemic caused by HPAIV H5N8 clade 2.3.4.4 group B (subsequently called H5N8B) was the most severe one reported in Germany and Europe so far. The epidemic started in Germany in November 2016, ended in May 2017, and was accompanied by local mass mortality events in wild birds affecting primarily water birds and scavenging birds [6,7]. Phylogenetic analysis of the viruses isolated from wild water birds suggested multiple independent incursions of reassortant viruses of at least five distinct genotypes [8]. In contrast to the 2014/2015 HPAI 2.3.4.4 group A viruses, which were sporadically found in apparently healthy wild birds and caused low mortality in experimentally infected ducks [9–11], H5N8B was shown to express augmented virulence for waterfowl, but low zoonotic potential [12,13].

Several experimental studies with different water bird species and intensified active wild bird surveillance have shown that dabbling ducks may play an efficient role in the maintenance and dissemination of low pathogenic avian influenza viruses (LPAIV), but also of H5-HPAIV, typically associated with lower mortalities as compared to other duck species [12,14–17]. The mallard duck (*Anas platyrhynchos*) is a species, for which a high incidence of avian influenza A virus (AIV) infections has repeatedly been reported. The pivotal role of mallard ducks as a reservoir for LPAIV has been explained by the feeding and migratory behaviour of dabbling ducks and the primarily fecal-oral transmission route of LPAIV [18]. The global distribution and abundance of this duck species certainly contributes to this.

Sentinel surveillance has been described as an efficient tool within active wild bird surveillance and for studying aspects of the ecobiology of AIV in dabbling ducks [19]. Since 2006, we have been continuously keeping mallard ducks as sentinels for circulating AIV. The ducks were held in an aviary located at the shallows of the Baltic coast in Germany in close contact to wild waterfowl and migratory birds [20]. During more than one decade of fortnightly testing for AIV infection in these sentinel mallard ducks as described earlier [20], HPAIV has never been detected, although H5N8B was circulating in wild ducks and gulls in the same geographical area in 2016/2017. Instead, multiple LPAIV infections of individual ducks were detected. Ducks that hatched in May 2017 had undergone natural infection with H1N3, H3N8, H4N6, H5N3 and H11N9 in the above-mentioned aviary. In November 2018, seven of these LPAIV pre-exposed sentinel mallard ducks were selected for an infection experiment with H5N8B. All ducks tested were strongly positive in the Nucleoprotein (NP)-antibody-ELISA 28 days and 1 day prior to the infection experiment.

Here we evaluated the role of mallards in the transmission of H5N8B after natural exposure to LPAIV (LPAIV + HPAIV H5N8B) targeting the following questions:

- Are naturally LPAIV pre-exposed mallard ducks susceptible for H5N8B infection, and which clinical signs and pathological lesions do they exhibit?
- How long do pre-exposed mallards shed virus and is shedding sufficient to infect co-housed naïve (susceptible) juvenile ducklings?
- How much virus is detectable in feces and water and what are the peak titers?

In addition, the variance in dynamics after re-infection of surviving ducks with the homologous clade 2.3.4.4B virus (H5N8B + H5N8B) was studied to answer the question if homologous antibodies in mallards are fully protective to efficiently prevent virus shedding.

In our study, we aimed at gaining insights into the pathobiology of naturally AIV pre-exposed mallard ducks challenged with H5N8B virus – proposed as a highly virulent duck virus during the 2016/2017 epidemic [12].

## Materials and methods

### Ethics statement

The animal experiments were approved by the State Office of Agriculture, Food safety, and Fishery in Mecklenburg-Western Pomerania under the registration number LALLF MV 7221.3-1.1-025/18 and LALLF MV/TSD/7221.3-2-006/19 which includes the approval and control of the local veterinary authority of Greifswald municipality to keep mallard ducks for scientific purposes. All procedures were carried out in the approved biosafety level 3 facilities of the Friedrich-Loeffler-Institut, Isle of Riems, Germany.

### Virus origin and propagation

The HPAI H5N8 clade 2.3.4.4B virus (H5N8B) strain (A/tufted duck/Germany/AR8444-L01987/2016) originated from the 2016/2017 epizootic in Germany and was used in this study. The virus was passaged one time and titrated using specific pathogen-free (SPF) 10 day-old embryonated chicken eggs (ECE). Infectivity titers are expressed as mean embryo infectious doses (EID_50_/ml).

### Animals

*Target group*: Seven 18-months old sentinel mallard ducks used in our study hatched in May 2017. They were bought from a local small-scale mallard breeder and tested seronegative against AI when purchased. All mallards were kept in the sentinel aviary of the FLI for 17 months. During that time, active infection with H1N3, H3N8, H4N6, H5N3, and H11N9 was detected in the group by real-time RT–PCR virus detection and subtyping [21]. We assume that all ducks had contact with those viruses. The sampling schedule was every fortnight; therefore, we could not detect the viruses in each of the mallards at the time-point of sampling. Serologically, all mallards were positive by ELISA for NP-specific antibodies. Sera were not tested by HI since homologous isolates could not be grown from the samples obtained. In the following, these ducks are named “seropositive mallards”.

*Infection Control group*: Seven 14-months old AI-seronegative Pekin ducks (*Anas platyrhynchos domesticus*) were selected as synspecific equivalents to mallards (“seronegative Pekin ducks”). The ducks hatched at the beginning of September 2017 and were raised in a commercial duck farm. Their seronegative (naïve) status regarding AIV was confirmed by NP blocking ELISA at the FLI before use in the animal experiment.

*Contact group*: Five-week old seronegative Pekin ducks (“contact ducklings”) were obtained from the same commercial farm, from which also the control group was purchased. Four ducks served as contact birds for each group and experiment, respectively. Their seronegative (naïve) status regarding AIV was confirmed by NP-ELISA test at the FLI before they were employed in the animal experiment.

For the experiments, the mallard and Pekin duck groups were housed in different stables of approximately 30 m^2^, where they could roam freely. Duck feed (pellets and wheat) and water was provided *ad libitum* and replenished at least once a day.

Each group had access to a (i) fresh water pool of 300 l, (ii) drinking trough with a volume of 14 l, and (iii) paddling pool of 20 l of water. Water samples were daily taken from each of the water sources before the water was changed.

### Experimental design

Experiments were conducted in November and December 2018

#### Experiment 1: LPAI-pre-exposed + HPAIV H5N8B

The ducks of the target group (*n* = 7) and the infection control group (*n* = 7) were nasally inoculated using a dose of 10^6^ EID_50_ HPAIV H5N8. After 48 h, 4 non-infected 5-week old seronegative Pekin ducklings served as sentinels (contact group) for each duck group. Daily swab samples from the oropharynx (OP) and cloaca (CL) were collected from all infected birds until 14 days post infection (dpi) and contact duckling 12 days post contact (dpc) into Eppendorf safe lock tubes with serum-free cell culture medium supplemented with antibiotics and fungicide according to the diagnostic manual for Avian Influenza [22]. Blood was taken for serological assays on 2, 14, 23 and 34 dpi. Furthermore, water samples were daily collected. Fecal matter was taken randomly from the ground by a swab from 3 dpi onwards and analyzed for the presence of viral RNA (vRNA) and replication competent virus.

#### Experiment 2: HPAI + HPAIV H5N8B (homologous re-Infection)

After 21 dpi, surviving animals of the target group (6 mallards) and the only surviving contact Pekin duckling of the target group were challenged with HPAIV H5N8B a second time in the same barn. The barn as well as the pool and water troughs were cleaned every day. Similar to experiment 1, four 4-week old Pekin ducks served as sentinels (contact group) from 2 dpi and viral shedding was examined by daily OP and CL swabbing as well as water and feces collection. As an infection control, 3 adult seronegative Pekin ducks were infected in a separate barn and sampled as described above. Feces and water samples were also taken.

The ducks were monitored and scored every 24 h until 14 dpi and for further 13 days if re-infected. The health status was scored as follows: 0 (normal); 1 (sick); 2 (severely sick) and 3 (dead). “Sick” birds showed only one of the following signs whereas “severely sick” animals showed more than one: Respiratory involvement, ruffled feathers, depression, diarrhea, cyanosis of the feet or mucosa, edema of the face and head, nervous signs. Moribund birds reaching humane termination criteria were permanently drowsy and recumbent, could not be urged to move or showed severe dyspnoeic movement of the sternum. Such ducks were humanely killed by use of isoflurane and subsequent cutting the *Arteria carotis* and registered as “3” (dead) the day after. Dead birds were scored as 3 on each of the remaining daily observations after death until the end of the observation period. After 14 days of the observation period, the sum of the observations in each category was divided by the total number of observations (average clinical score).

All surviving ducks were euthanized on 34 dpi and finally bled for serum preparation.

### Macroscopical, pathohistological and immunohistological investigations

A complete necropsy was performed under biosafety 3 level conditions according to internal standard guidelines. Samples were collected from brain, heart, lungs, spleen, liver, kidneys, pancreas and duodenum, fixed in 4% neutral buffered formaldehyde for more than 21 days, processed and embedded in paraffin wax. Hematoxylin and eosin stained sections were evaluated for histopathological lesions using a light microscope, and the severity of parenchymal necrotizing inflammation, as well as lymphatic necrosis, apoptosis and/or depletion in the lymphatic organs was scored on an ordinal 4-step scale (0 = unchanged, 1 = mild, 2 = moderate, 3 = severe). Immunohistochemistry was employed to detect influenza A virus matrix protein using the avidin–biotin-peroxidase-complex method (Vectastain PK 6100; Vector Laboratories, Burlingame, CA, USA) with citric buffer (10 mM, pH 6.0) pretreatment, a monoclonal antibody (mAb) directed against an epitope of the influenza A virus matrix protein (ATCC clone HB-64), 3-amino-9-ethylcarbazol chromogen (Agilent Technologies, Santa Clara, CA, USA), and hematoxylin counterstain [23]. Validated positive and negative archival tissues, as well as replacement of the specific antibody by an IgG directed against a surface epitope of chicken lymphocytes (clone T1) [24]. The distribution of parenchymal influenza A virus matrix protein was evaluated on an ordinal 4-step scale (0 = none, 1 = focal/oligofocal, 2 = multifocal, 3 = coalescing/diffuse).

### Virological investigations

Swabs and tissue samples of the individual animals were re-suspended in 1 ml serum-free medium supplemented with antibiotics and fungicide. A single stainless-steel bead (5 mm) was added for organ samples and homogenized in a 2 ml collection tube for 2 min in a TissueLyser instrument (Qiagen, Hilden, Germany). Viral RNA was extracted from swab and fecal fluid, water samples and organ homogenates using the NucleoMag®VET Kit (Macherey-Nagel GmbH & Co. KG, Düren, Germany, Lot 18081003) according to the manufacturer’s instructions. The presence of RNA of the influenza A virus matrix (M) gene was confirmed by quantitative real-time RT–PCR (RT-qPCR) (AgPath-ID One-step RT–PCR Kit, Ambion, Austin, TX, USA, Lot 1802220, 1805222) following the modified protocol of Spackman et al. [25]. An additional reverse primer was added to accommodate detection also of the new human pandemic H1N1 virus of 2009 [26]. H5-specific RNA was examined using primers and probes as recommended by the European Union method [27]. Samples with a cycle of quantification value (Cq) of 39.5 (limit of detection, lod) or higher were regarded as negative. The lod is the lowest Cq value likely to be reliably distinguished from RNA internal controls (RICs), which are always included in the RNA extraction and RT-qPCR analysis to fulfil QM standards. A standard curve for virus quantification was generated using extracted viral RNA from diluted HPAIV H5N8 suspensions with known infectivity titre by RT-qPCR targeting the M and H5 genes. RT-qPCR was conducted on a Bio-Rad platform using Bio-Rad C1000 Touch Thermal Cycler and Bio-Rad CFX96 Optical Reaction Module. To relate M- and H5-specific Cq-values to viral infectivity in the examined sample, Cq-values from these extracts were plotted on a standard curve linking infectivity with Cq-values.

### Serological investigations

Sera were treated at 56 °C for 120 min to inactivate complement and subsequently tested for the presence of anti-nucleoprotein (NP) antibodies using the competitive IDEXX AI Multi-Screen Ab ELISA kit (IDEXX, Maine, USA, Lot 7066) and the ID Screen® Influenza A Antibody Competition Multi-species ELISA (IDVET, Grabels, France, Lot D78) according to the kit protocols. Furthermore, the ID Screen® Influenza H5 Antibody Competition kit (IDVET, Grabels, France, Lot C99) was used for the detection of H5-specific antibodies. Sample to Negative (S/N) values were calculated and S/N values below 45% were regarded as positive for the ID Screen® Influenza A Antibody Competition Multi-species ELISA and S/N values below 50% were regarded as positive for the ID Screen® Influenza H5 Antibody Competition kit. The yolk of mallard duck eggs was diluted in 0.85% NaCl in a ratio of 1:4, frozen and de-frosted three times in total. After centrifugation at 3220 × g at 4°C for 20 min, the supernatant was harvested and processed as described for serum samples.

### Statistical analysis

Statistical analysis was performed using the R 3.4.1 software package (R Foundation for Statistical Computing, Vienna, Austria). Inferential statistics are intended to be exploratory (hypotheses generating), not confirmatory, and are interpreted accordingly. I.e. *p*-values are interpreted in Fisher's sense, representing the metric weight of evidence against the respective null hypothesis of no effect. Neither a global significance level nor local levels are determined. *P*-values are considered noticeable in case *p* ≤ 0.05 and highly noticeable in case *p* ≤ 0.01. Noticeable findings are primarily meant for generating new hypotheses that need to be verified in further experiments or in observational studies. Further statistical analyses were carried out using the GraphPad PrismTM software (v 8.1.0) (La Jolla, CA, USA). Continuous (oral and cloacal shedding) and ordinal data (clinical scores) are shown as absolute values in line graphs for each duck and time point for the whole observation period. Mortality was also analyzed by time-to-event methods, i.e. Kaplan-Meier plots. Overall survival was calculated starting from the day of first and second infection until death with censoring of ducks alive at the end of the observation period.

## Results

### Experiment 1: natural LPAI-pre-Exposure + HPAIV H5N8B challenge infection

#### Clinical disease: mild in seropositive mallards, fatal in seronegative ducks and contact ducklings

**Figure 1.**
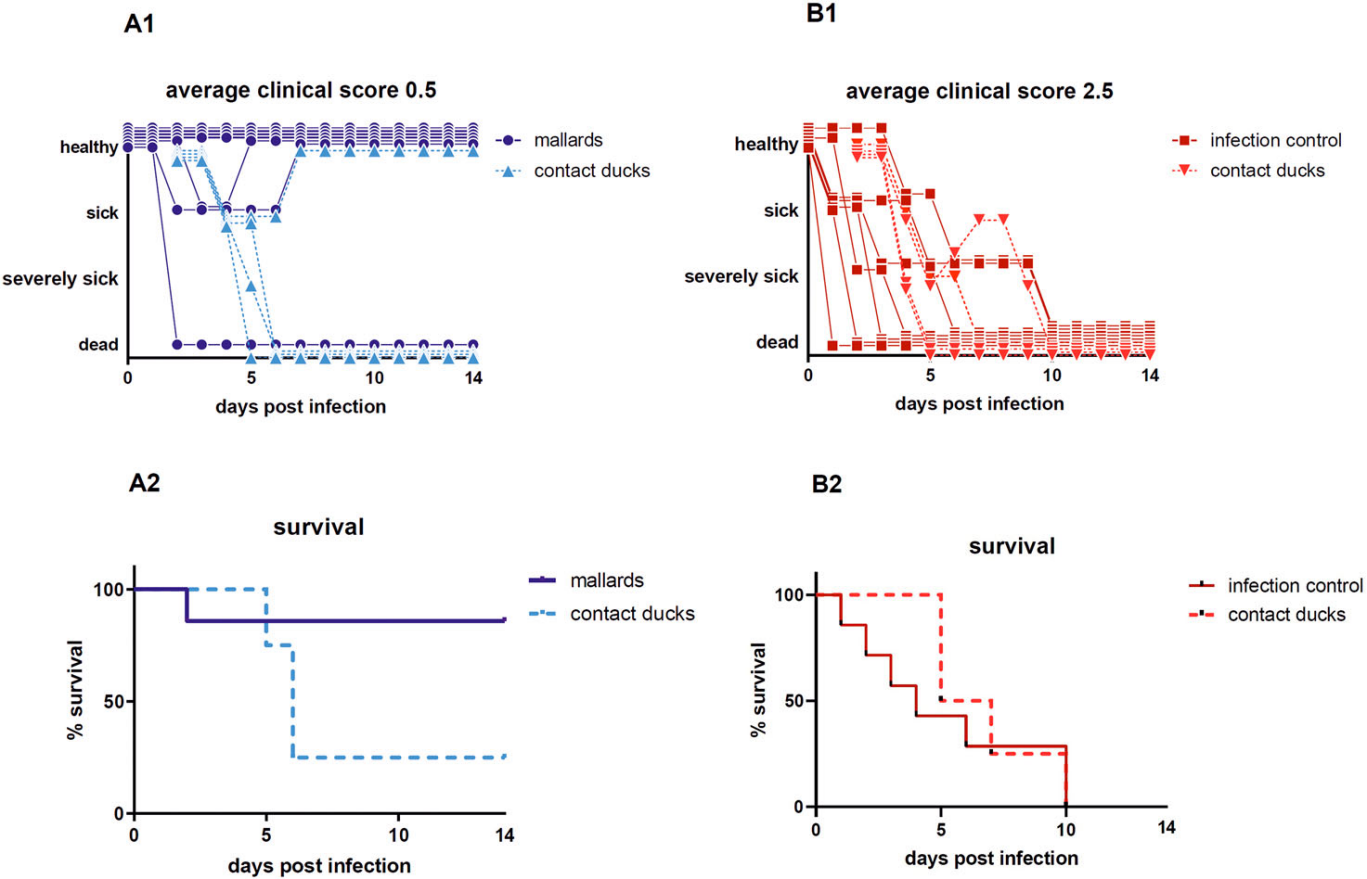
Clinical score and survival rate of the target group (seropositive Pekin ducks; B1; B2) and the control group (seronegative Pekin ducks; B1; B2). Of the 7 mallards, only 2 ducks appeared depressed for 2–3 days, but one died, while 3 of 4 contact ducklings died until 4 days post contact. In the seronegative Pekin duck group, all 7 ducks, including all 4 contact ducklings, developed severe clinical disease and died within 9 days post infection/contact. Three birds that were humanely killed were registered as “3” (dead) the day after.

Upon infection with HPAIV H5N8B the average clinical score for seropositive mallards was 0.5 in comparison to the adult seronegative Pekin ducks with a much higher score of 2.5 (Figure 1(A1, B1)). Mallards showed some mild, transient, greenish diarrhea between 2 and 4 dpi along with slight lethargy. After this period, 6 of 7 mallards recovered completely and were clinical healthy for the rest of the experiment resulting in a case fatality rate of 14%. One mallard duck died peracutely without any clinical signs and was found dead in the morning of day 2 after infection (supplemental figure). The case fatality rate in seronegative Pekin ducks (*n* = 7) was 100%. They developed strong greenish diarrhea from day 2 dpi and became lethargic. Sick Pekin ducks that survived the acute phase developed neurological symptoms within 4 days, i.e. head shaking, tremor of the body, torticollis, slow walking or disorders in balance and were euthanized 9 dpi at the latest (Figure 1). The mortality among the contact ducklings in the mallard group was 75% (3 of 4 dead), whereas all contact ducklings in the seronegative Pekin duck group died within 8 days post contact (dpc) (Figure 1(A2, B2), supplemental figure).

#### Viral shedding: virus concentration in oral swabs was higher than in cloacal swabs for up to seven days in seropositive mallard ducks, sufficient to transmit virus to contact ducklings

**Figure 2.**
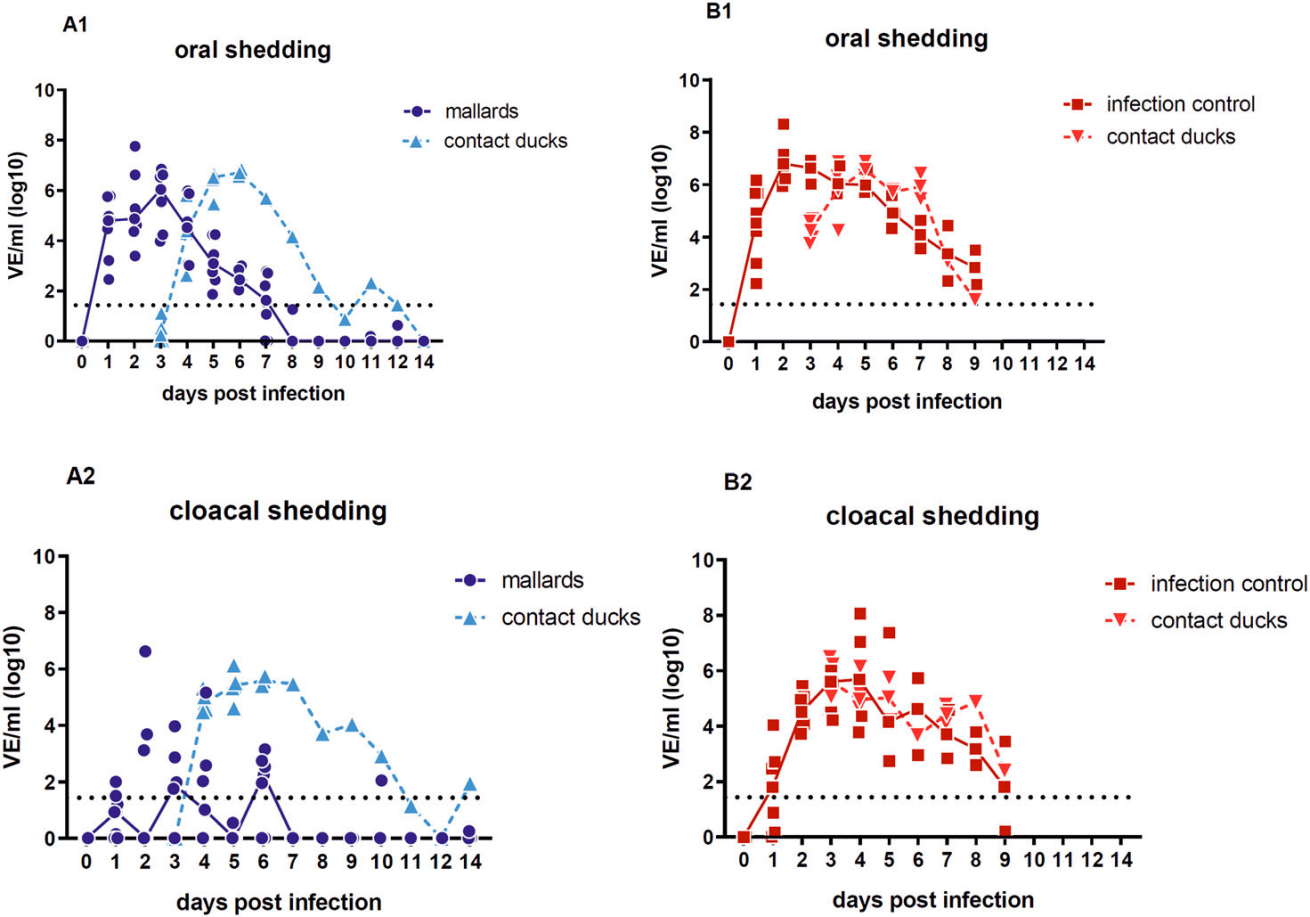
Oral versus cloacal shedding in virus equivalents per ml (VE/ml) per log_10_ derived from H5-specific RTqPCR. A: target group (seropositive mallard ducks) and contact ducklings; B: infection control group (seronegative Pekin ducks) and contact ducklings. In the infection control group, the shedding was terminated after 9 dpi due to the death of all ducks in the group. Individual results of detected RNA copy numbers are given as virus equivalents (VE), calculated using a standard curve with data from each PCR run. The lines connect the medians calculated for each day post infection. The limit of detection is at 10^1.43^ VE/ml (dotted line).

Virus shedding started 1 dpi and higher virus equivalents were detected in oropharyngeal than in the cloacal samples (Figure 2(A1, B1)). Concentration of virus shed by the cloaca in the seropositive mallards was reduced in comparison to all other ducks. In average, viral shedding of the seropositive mallards was lower than that of all other seronegative Pekin ducks including the contact ducklings. For the target group (seropositive mallards), oral viral shedding after 4 dpi was less than 10^4.5^ virus equivalents (VE)/ml, and the peak of oral vRNA shedding was on 3 dpi with 10^6.85^ VE/ml (Figure 2(A1, A2)). In line with the shedding data, virus was efficiently transmitted to the contact ducklings (triangles in Figure 2). The surviving contact duckling in the target group shed virus until 9 dpc.

For the infection control group (seronegative Pekin ducks) the viral shedding remained on a high titer level between 10^6^ and 10^6.91^ VE/ml on 3 dpi with the highest shedding levels at day 2 for oral and at day 4 for cloacal swabs (>10^8^ VE/ml). Furthermore, the viral shedding found in oral swabs from the ducklings that had contact to the target and control group was comparable 3 dpc and reached 10⁵^.^⁴² to 10⁶^.^⁸⁶ VE/ml, respectively. The level of shed virus in oral swabs from the contact ducklings was in line with the infection control. Finally, the shedding declined to 10^2.8^ VE/ml until 9 dpi/7 dpc before all seronegative Pekin ducks and ducklings were dead (Figure 2(B1, B2)). Furthermore, prior to the start of the *in vivo* study, the adult Pekin ducks (all female) laid unfertilized eggs after they were placed in the stable prior to the start of the study. The last egg was laid 2 dpi and 10^3.9^ VE/ml were detected in the white of the egg.

In addition, blood taken 2 dpi of 4 seronegative Pekin ducks and 2 seropositive mallard ducks tested positive for H5 RNA with titers ranging from 10^2.2^–10^5.8^ VE/ml indicating the start of a systemic infection.

#### Challenge virus (HPAIV H5N8B) in environmental samples: detection of infectious virus in water samples up to 9 dpi

The sampled feces from the seronegative Pekin ducks were vRNA positive from the first day of sampling (i.e. 3 dpi) until the last duck was euthanized on 9 dpi. The titers peaked 3 dpi at 10^7^ and 5 dpi at 10^6.2^ VE/ml. In contrast, in the target group, shorter virus shedding and lower vRNA titers were found in feces (4–8 dpi) with highest titers of 10^5.1^ and 10^5.7^ VE/ml on 5 and 7 dpi, respectively.

From 2 dpi onwards, water samples were strongly positive (up to 10^4.8^ VE/ml) for vRNA until 8 dpi in the infection control Pekin duck group and until day 7 pi and once again 9 dpi in the target group. In summary, the quantities of vRNA in water samples were comparable over time. Finally, virus could be isolated and propagated in ECEs from water samples within the mallard group taken 2 dpi (pool) and 6 dpi (trough) (Table 1).

**Table 1.** Quantity of viral RNA in different water samples from the two groups (mallards and infection control Pekin ducks), presented as virus equivalents per ml (VE/ml) per log_10_ (H5-specific RTqPCR).

		0 dpi	1 dpi	2 dpi	3 dpi	4 dpi	5 dpi	6 dpi	7 dpi	8 dpi	9 dpi	10 dpi	11 dpi	12 dpi	14 dpi
	H_2_O	vRNA/VI	vRNA/VI	vRNA/VI	vRNA/VI	vRNA/VI	vRNA/VI	vRNA/VI	vRNA/VI	vRNA/VI	vRNA/VI	vRNA/VI	vRNA/VI	vRNA/VI	vRNA/VI
Mallards	Pool (300l)	−/n.d.	−/n.d.	**4.09/+**	4.34/−	3.86/−	2.55/−	2.98/−	2.45/n.d.	−/n.d.	1.91/n.d.	−/n.d.	−/n.d.	−/n.d.	−/n.d.
	Trough (14l)		−/n.d.	−/n.d.	−/n.d.	2.26/n.d.	4.18/−	**4.51/+**	2.66/n.d.	−/n.d.	−/n.d.	−/n.d.	−/n.d.	−/n.d.	−/n.d.
	Paddling (20l)					4.20/−	3.65/−	3.71/−	−/n.d.	−/n.d.	−/n.d.	−/n.d.	−/n.d.	−/n.d.	−/n.d.
Infection Control	Pool (300l)		−/n.d.	1.65/n.d.	3.73/−	4.47/−	4.81/−	4.26/−	2.63/n.d.	2.11/n.d.	−/n.d.				
	Trough (14l)		−/n.d.	3.88/−	3.99/−	3.97/−	2.68/n.d.	3.88/−	1.52/n.d.	−/n.d.	−/n.d.				

Notes: In bold: Infectious virus could be propagated in embryonated chicken eggs.

Dpi, days post infection; viral RNA (vRNA) presented as VE/ml per log_10_; VI: Virus isolation; −negative; + positive; n.d. not done.

#### Serology: seroconversion or increased H5 antibody titers were associated with survival in ducks

**Figure 3.**
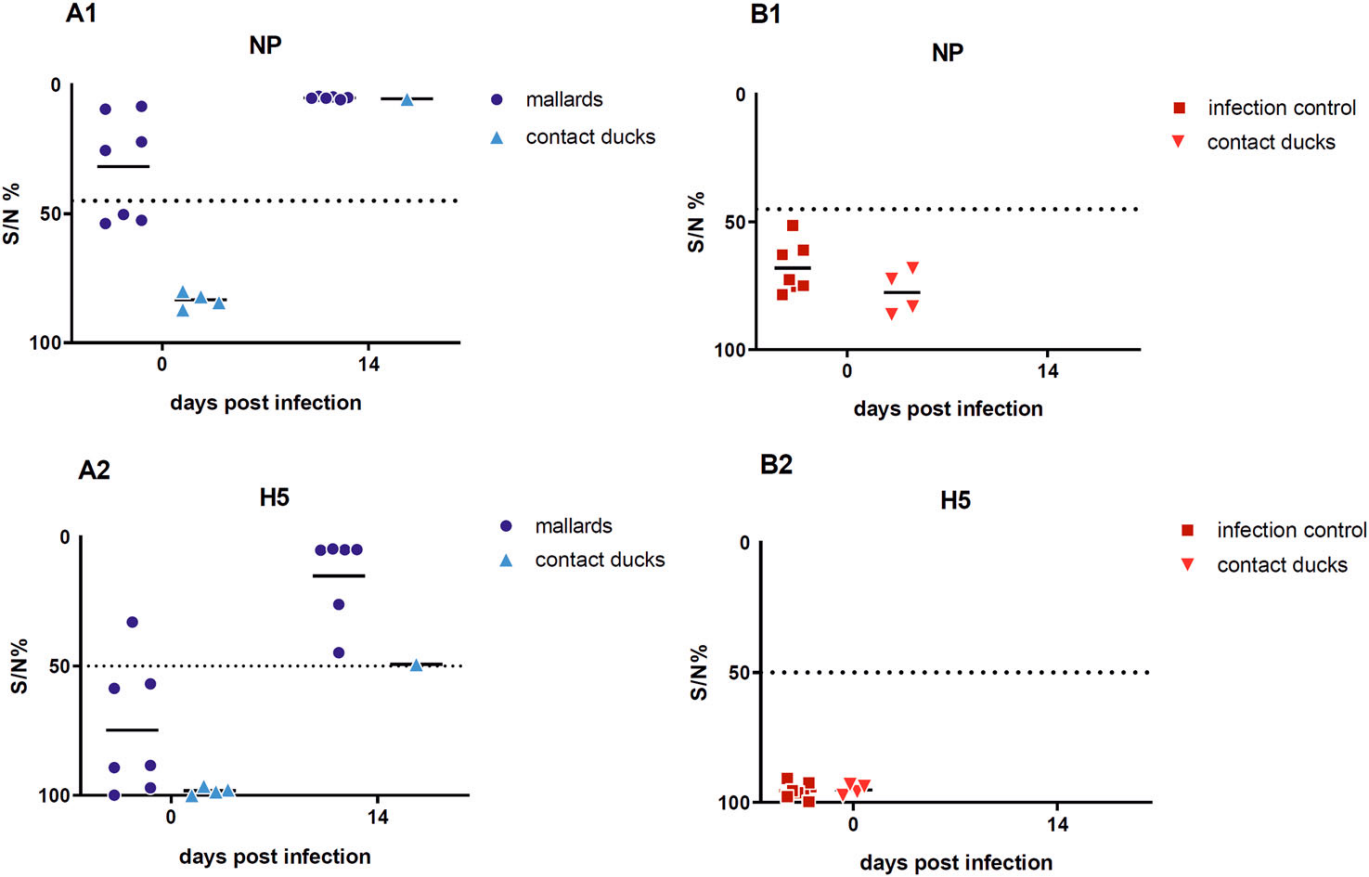
Results of the competitive ELISA kits showing NP- and H5-specific seroconversion of the seropositive mallards and one contact duckling that survived a challenge infection with HPAIV H5N8B (A) and of the seronegative Pekin ducks serving as infection control group with contact ducklings (B). Lines represent the median. Inhibition of less than 45% for NP and of 50% for H5 is regarded as seropositive. NP = Nucleoprotein.

All surviving seropositive mallards showed increased NP-specific antibody levels on 14 dpi as compared to the serological status prior to the infection (Figure 3). H5-specific antibodies in all surviving ducks were also confirmed using the ID-Screen ELISA. One duck had H5-specfic antibodies tested in H5-ELISA prior to the H5N8B virus infection (Figure 3(A2)), most likely as a result with a preceding field infection with LPAIV H5N3.

#### Pathology and tissue viral load: hepatotropism was observed in all ducks that died within 5 dpi/dpc

**Figure 4.**
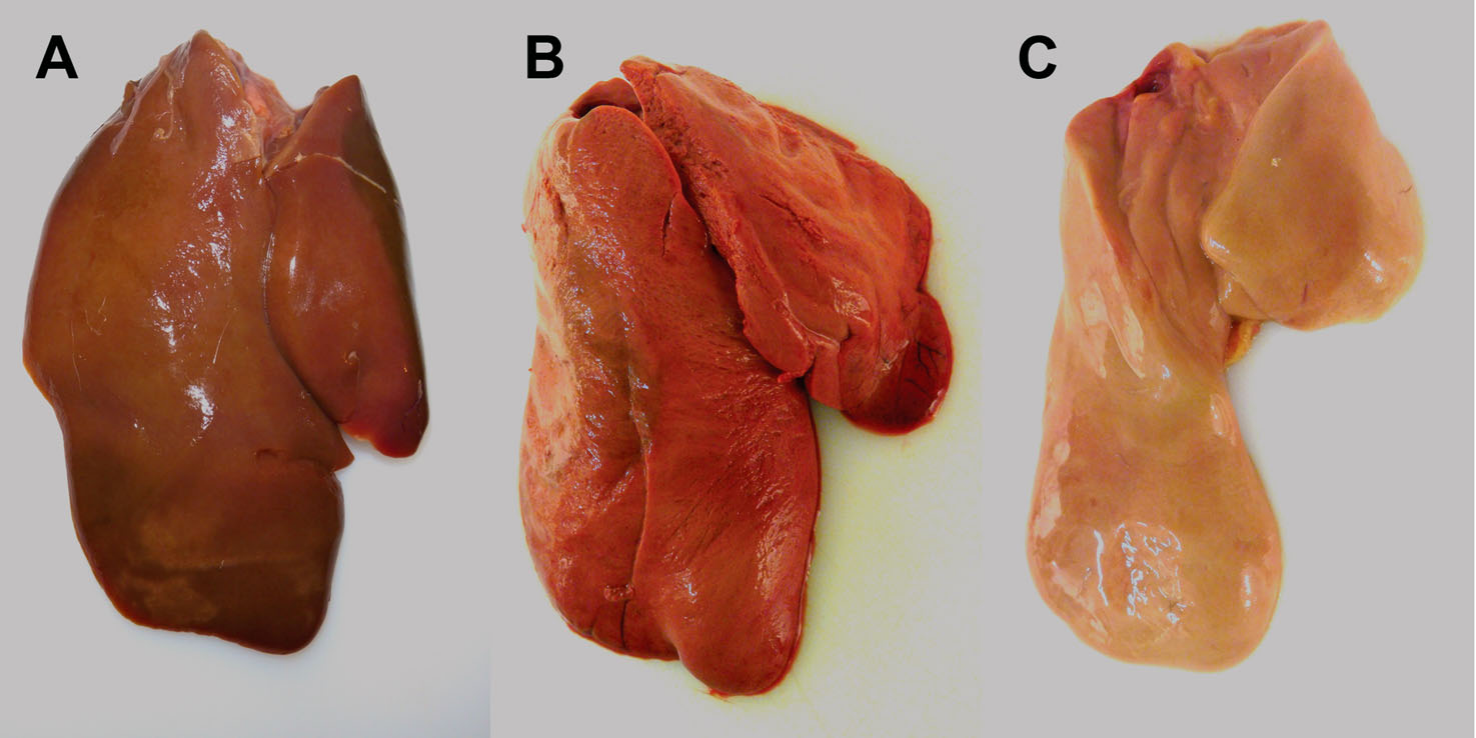
Macroscopic findings in the livers of experimentally H5N8B-infected ducks. (A) Seronegative Pekin duck, H5N8B-infected, euthanized 9 dpi due to neurological symptoms, liver. Macroscopically normal, brown-red, acutely-angled liver without immunohistochemically-detectable hepatocellular influenza A virus matrix protein. (B) Pekin contact duckling in the mallard group, died 4 dpc, liver. Swollen, brick-red-colored, friable liver with rounded edges, interpreted as severe, acute, diffuse, necrotizing hepatitis with immunohistochemically-detectable hepatocellular influenza A virus matrix protein. (C) Seropositive mallard, H5N8B-infected, clinically normal, 14 dpi, liver. Swollen, beige, greasy liver with rounded edges, interpreted as moderate, acute, diffuse hepatocellular lipidosis (background pathology) without immunohistochemically detectable hepatocellular influenza A virus matrixprotein.

**Figure 5.**
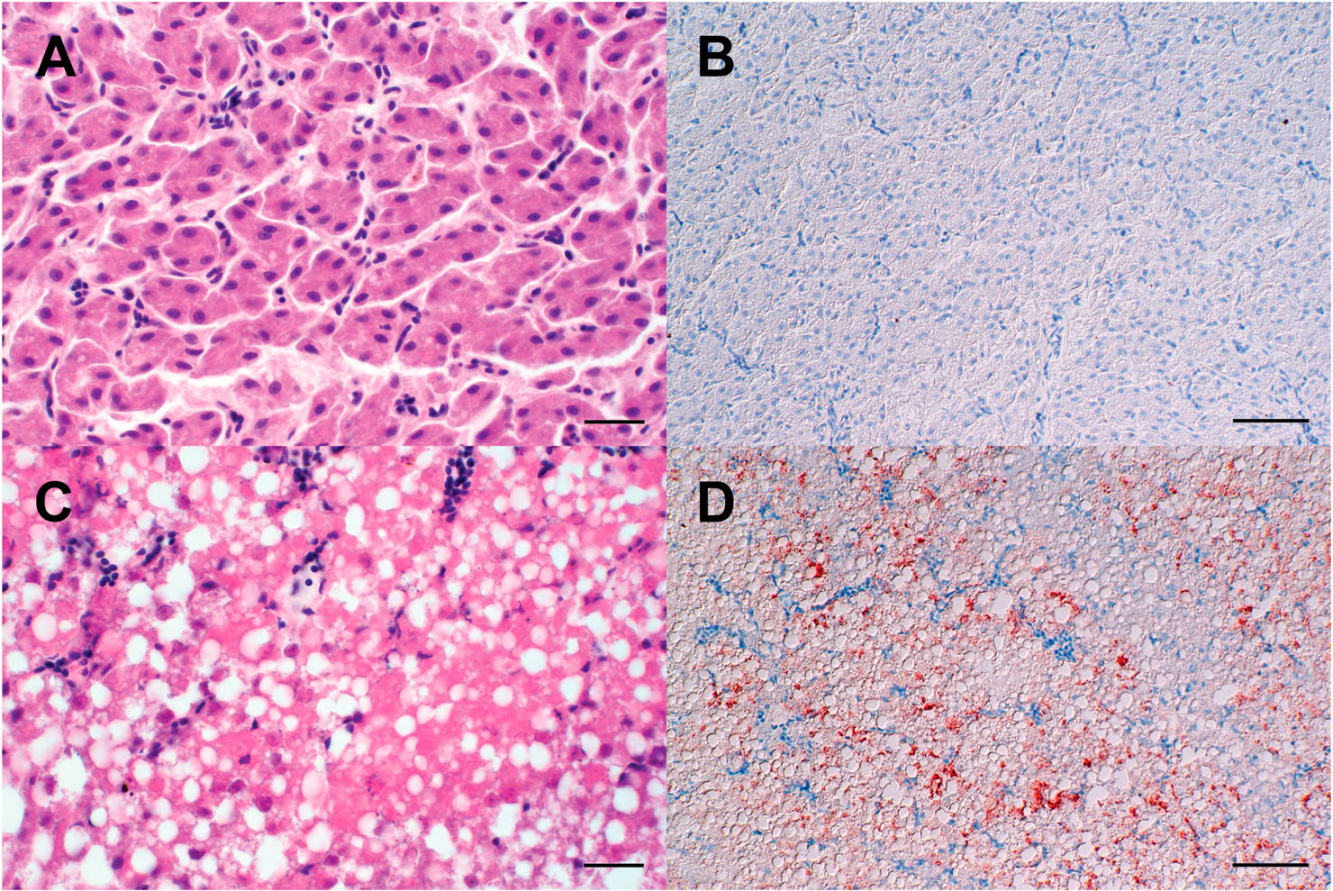
Light microscopic finding in the livers of experimentally H5N8B-infected ducks. (A, B) Seropositive mallard, H5N8B-infected, clinically normal, 34 dpi, liver. (A) No obvious findings. (B) Lack of immunohistochemically-detectable hepatocellular influenza A virus matrixprotein antigen. (C, D) Pekin duck, contact animal, died 4 days post contact, liver. (C) Marked hypereosinophilia, hepatocellular vacuolation, membraneous rupture and nuclear pyknosis, karyorrhexis and lysis interpreted as severe, acute, coalescing to diffuse necrotizing hepatitis. (D) Immunohistochemistry reveals coalescing intrahepatocytic, intracytoplasmic and intranuclear influenza A virus matrix protein. (A, C) Hematoxylin-eosin, (B, D) Immunohistochemistry using the avidin-biotin-peroxidase-complex method with a monoclonal antibody against influenza A virus matrix protein (ATCC clone HB-64), 3-amino-9-ethylcarbazol chromogen (redbrown) and hematoxylin counterstain (blue). (A, C) bars = 20 μm. (B, D) bars = 50 μm.

**Figure 6.**
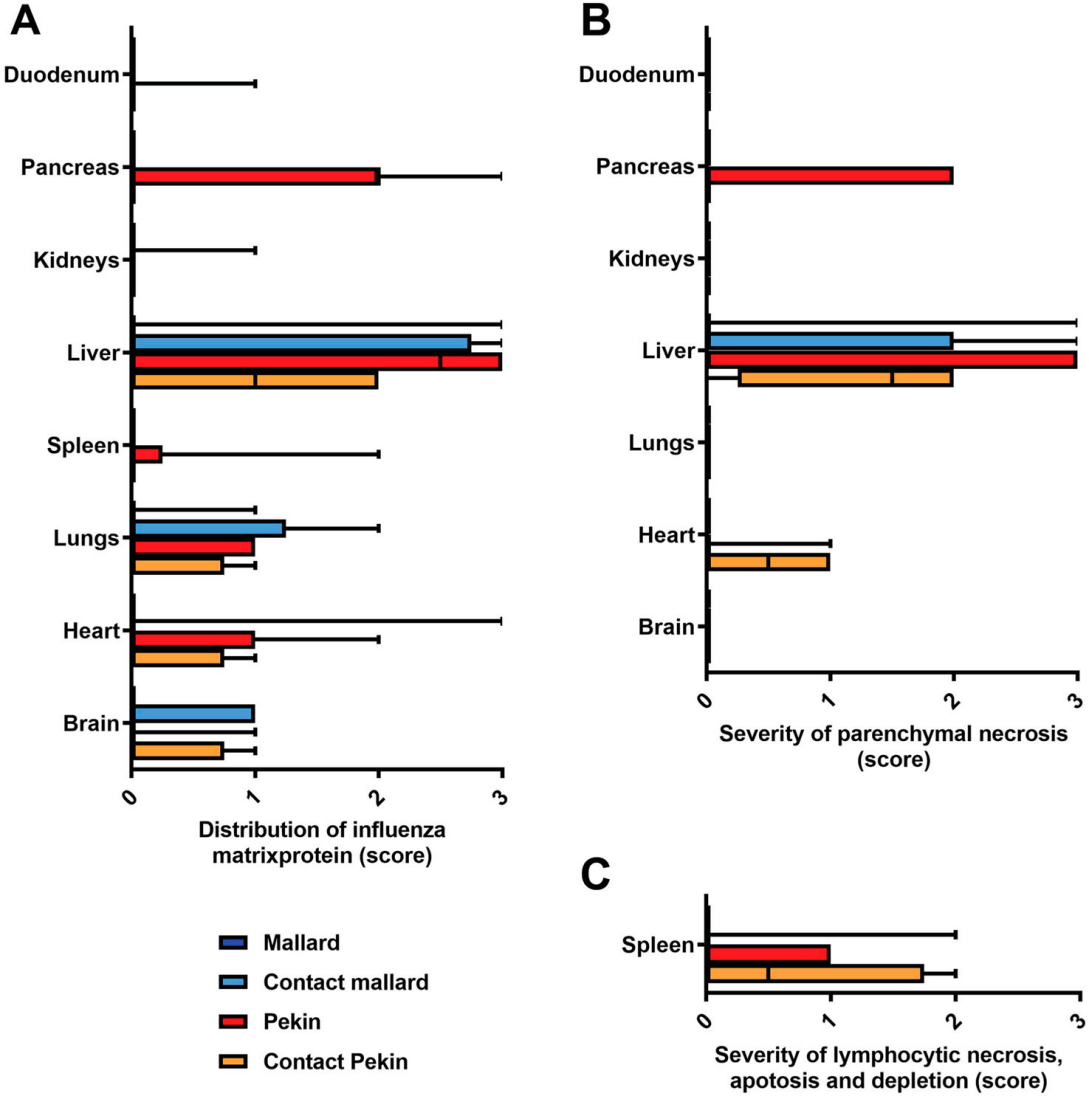
Light microscopic assessment of virus tropism (A) and lesion profile (B,C) revealed a predilection of the liver for H5N8B induced lesions in contact mallards, Pekin ducks, and contact Pekin ducks. (A) The distribution of influenza virus matrixprotein was evaluated on an ordinal scale: 0 = none, 1 = focal/oligofocal, 2 = multifocal, 3 = coalescing/diffuse, and revealed a systemic virus infection in many contact mallards, Pekin ducks, and contact Pekin ducks as compared to the mallards. (B, C) The severity of parenchymal necrosis (B), or lymphocytic necrosis, apoptosis and depletion in the spleen (C) was evaluated on an ordinal scale as follows: 0 = unchanged, 1 = mild, 2 = moderate, 3 = severe. Box-and-whisker plots display the median, the quartiles as well as min and max of the semiquantitative scores.

The majority of seronegative Pekin ducks and the contact ducklings died between 2 and 6 dpi/dpc (acute phase of disease) with macroscopic and histopathologic signs of acute necrotizing hepatitis of a variable and mostly severe grade (Figures 4(B), 5(C), 6), and with intralesional, intrahepatocytic influenza A virus antigen (Figure 5(D)). Some of these animals exhibited concurrent diffuse hepatocellular lipidosis, which can hide concurrent low-grade hepatocellular necrosis at gross inspection. Furthermore, some Pekin ducks displayed mild to moderate, acute, multifocal necrotizing pancreatitis with intralesional, intraepithelial influenza A virus matrixprotein as a second organ manifestation of major relevance for the clinical outcome (Figure 6(B, C)). Endotheliotropism was never detected within the ducks by immunohistochemistry. Viral loads as quantified by RT-qPCR were most prominent in the liver followed by the lungs, brain, nasal conchae, intestine, and quill (Table 2). The three Pekin ducks that were euthanized after they had developed central nervous symptoms did not show necrosis of the liver (Figure 4(A), supplemental file) and had lower viral loads in all organs compared to the ducks that died earlier.

**Table 2.** Clinical symptoms, gross lesions and virus distribution in tissue samples of HPAIV H5N8B infected groups and contact animals.

Group	Infection	Clinical symptoms							Pathology: gross lesions			Virus detection by PCR						
	HPAIV H5N8B	Green diarrhoea	Depression	Conjunctivitis	Nasal/eye discharge	Facial edema	Neurological symptoms (imbalance, shaky motion, torticollis)	Death	Macula feet	Lung hyperemia	Liver necrosis	Liver	Lungs	Cloaca	Intestine	Nasal conchae	Brain	Quill
Seropositive mallards	0 dpi + 21 dpi	2/7	0/7	0/7	1/7	0/7	0/7	1/7	0/7	1/7	1/7	1/7 (++++)	1/7 (++++)	1/7 (++)	1/7 (++)	1/7 (++)	1/7 (+++)	1/7 (++)
Mallard contact ducklings	contact 2 dpi	0/4	3/4	1/4	2/4	0/4	0/4	3/4	1/4	0/4	2/4	3/4 (++++)	3/4 (+++)	3/4 (++)	3/4 (++)	3/4 (+++)	3/4 (+++)	3/4 (+++)
Mallard contact ducklings	contact 23 dpi	0/4	2/4	0/4	0/4	0/4	1/4	0/4	0/4	0/4	0/4	0/4	0/4	0/4	0/4	0/4	0/4	0/4
Seronegative Pekin ducks	0 dpi OR 21 dpi	4/10	5/10	0/10	3/10	2/10	4/10	10/10	1/10	4/10	7/10	10/10 (+++)	10/10 (+++)	10/10 (++)	10/10 (++)	10/10 (++)	10/10 (++)	10/10 (++)
Pekin contact ducklings	contact 2 dpi	1/4	2/4	0/4	0/4	0/4	2/4	4/4	1/4	2/4	3/4	4/4 (+++)	4/4 (+++)	4/4 (++)	4/4 (++)	4/4 (++)	4/4 (++)	4/4 (++)

Dpi, days post infection; dpc, days post contact; average Cq values: ++++, 15 < Cq <20; +++,20 < Cq < 25; ++,25 < Cq < 30; +,30 < Cq < 35

Neither gross nor histopathological lesions were obvious in any of the seropositive mallards that had survived the H5N8B challenge infection and were euthanized after 34 days (Table 2). The male mallard showed diffuse hepatocellular lipidosis without immunohistologically-detectable hepatocellular influenza A virus matrix protein (Figure 4(C)).

The single seropositive mallard that died within 48 h post infection presented a systemic infection with high H5N8 titers of 10^8.5^ VE/ml in the liver, 10^7.9^ VE/ml in the lung, 10^5.7^ VE/ml in the intestine, 10^5.3^ VE/ml in the nasal conchae, 10^7.1^ VE/ml in the brain and 10^5.9^ VE/ml in the quill. Light microscopy confirmed the gross suspicion of a severe, acute, coalescing to diffuse necrotizing hepatitis with diffuse intralesional, intrahepatocytic influenza A virus matrix protein, as well as concurrent diffuse hepatocellular lipidosis.

### Experiment 2: HPAI + HPAIV H5N8 (homologous re-Infection)

**Figure 7.**
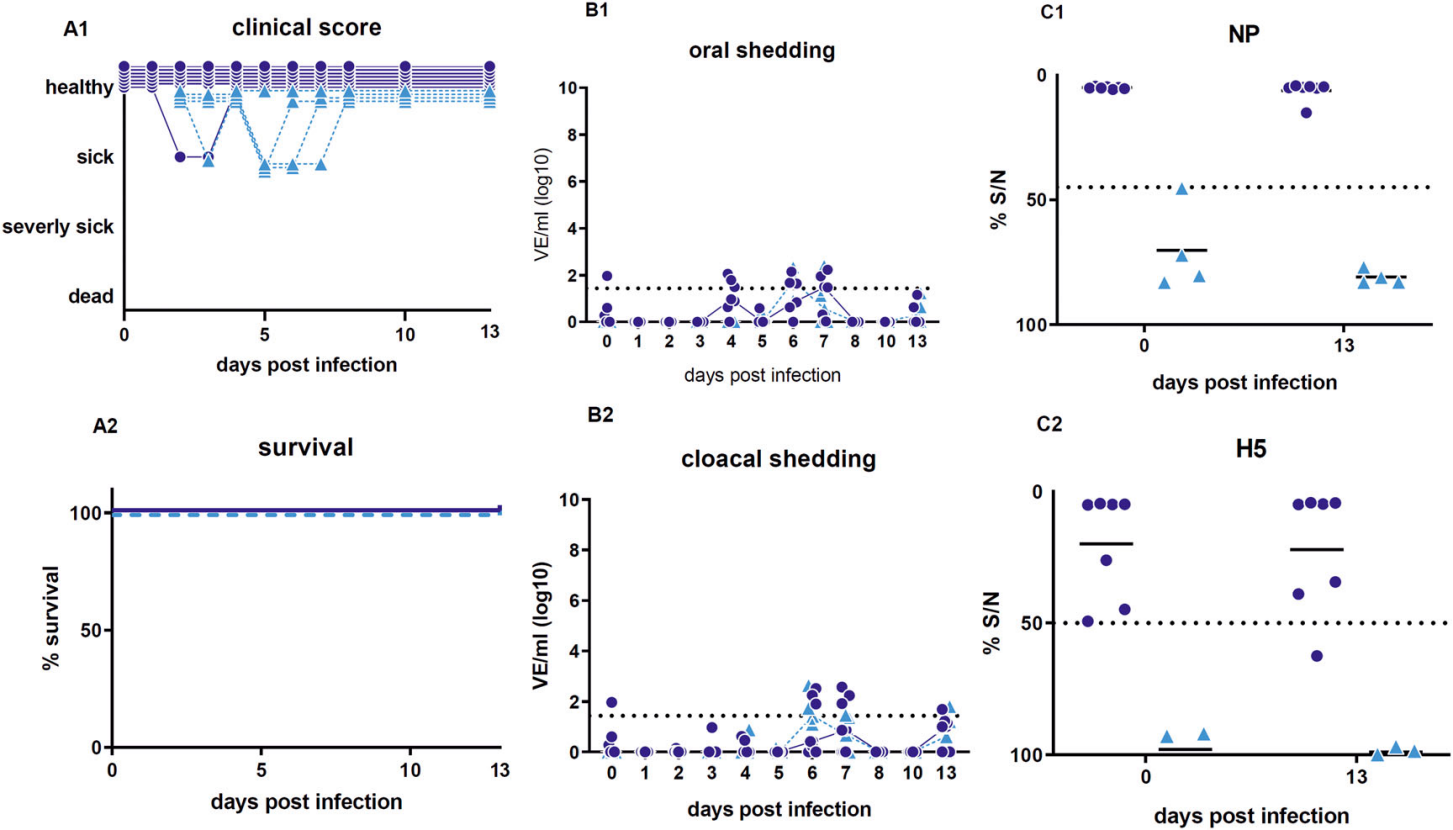
Survival rate and clinical score (A), viral shedding (B) and serological results (C) for homologously HPAIV H5N8 re-infected ducks (21 days after the first infection). B1+B2: Individual results of detected RNA copy numbers are given as virus equivalents (VE), calculated using a standard curve with data from each PCR run. The limit of detection is at 10^1.43^ VE/ml (dotted line). C1+C2: Results of competitive NP- and H5-specific ELISAs. Inhibition of less than 45% for NP and of 50% for H5 is regarded as seropositive. The lines in B and C represent the medians calculated for each day post infection. NP = Nucleoprotein.

Homologous re-infection of the surviving ducks (6 seropositive mallard ducks plus 1 surviving contact duckling from the same group, *n* = 7) resulted in full protection from clinical disease in the challenged ducks and a high antibody titer (Figure 7(C)). Naïve contact ducklings that were co-housed 2 dpi did not show any remarkable signs of disease. None of the ducks died. Three of the female mallards started laying eggs around 28 dpi. Minute vRNA shedding of 10^2.6^ VE/ml of one individual duck, i.e. a value at the limit of detection (10^1.4^ VE/ml), was recorded and none of the contact ducklings seroconverted (Figure 7). One mallard duck showed a decline in NP and H5-specific antibody titer on 34 dpi. The egg yolk of the mallard ducks had a high titer of NP and H5-specific antibodies at 34 dpi (NP %S/N 4.8 and 16.2 and H5%S/N 4.8 and 4.5, respectively).

Small amounts of vRNA were found in feces sampled on 3, 6 and 13 dpi. The titers were low and did not exceed 10^2.2^ VE/ml on day 6. In accordance with the results for the fecal samples, only on 3, 4 and 5 dpi very low levels of vRNA of up to 10^1.7^ VE/ml were detectable in water samples of the mallard group. The titers were just above the detection limit of the RTqPCR.

The 3 Pekin ducks of the infection control died peracutely at 3 or 4 dpi, confirming the clinical symptoms and pathohistological lesions and therefore, the validity of this challenge round (supplemental figure). The results for the fecal and water samples of the infection control group were in line with those obtained in the first H5N8B infection. Furthermore, infectious virus was propagated in ECE from a water sample with vRNA of 10^5^ VE/ml taken from the trough on 3 dpi from the infection control group (Table 3).

**Table 3.** Quantity of vRNA in water samples of different sources after the homologous infection.

		0 dpi	1 dpi	2 dpi	3 dpi	4 dpi	5 dpi	6 dpi	7 dpi	8 dpi	10 dpi	13 dpi
	H20	vRNA/VI	vRNA/VI	vRNA/VI	vRNA/VI	vRNA/VI	vRNA/VI	vRNA/VI	vRNA/VI	vRNA/VI	vRNA/VI	vRNA/VI
Mallards	Pool (300l)	−/n.d.	−/n.d.	−/n.d.	−/n.d.	−/n.d.	1.56/n.d.	−/n.d.	−/n.d.	−/n.d.	−/n.d.	−/n.d.
	Trough (14l)	−/n.d.	−/n.d.	−/n.d.	1.53/n.d.	1.74/n.d.	1.73/n.d.	−/n.d.	−/n.d.	−/n.d.	−/n.d.	−/n.d.
	Paddling (20l)	−/n.d.	−/n.d.	−/-	1.67/n.d.	1.42/n.d.	1.98/n.d.	−/n.d.	−/n.d.	−/n.d.	−/n.d.	
Infection Control	Pool (300l)		−/n.d.	3.27/−	2.69/n.d.	3.34/n.d.						
	Trough (14l)		−/n.d.	−/n.d.	5.14/+	4.27/−						

Dpi, days post infection; viral RNA (vRNA) presented as VE/ml per log_10_; VI: Virus isolation; −negative; +positive; n.d. not done.

## Discussion

Since the first incursion of goose/Guangdong lineage HPAIV H5N1 in Europe in 2006, we have been keeping mallards as sentinel ducks (with replacement of animals every two years at latest) in the shallows of the Baltic sea of northeastern Germany in an attempt to monitor circulating AIV. Although these ducks had infections with LPAIV, no HPAIV has yet been detected in them. In 2016, a novel reassortant HPAIV H5N8 2.3.4.4B has caused unprecedented mortality in wild water birds leading to an Eurasian-wide epidemic in wild birds and poultry [28]. In infection experiments using HPAIV H5N8B, the virus showed increased virulence plus neuro- and hepatotropism in Pekin ducks [12,29].

Between 2016 and 2018, the sentinel mallard flock was naturally exposed to several LPAIV subtypes that were circulating in the wild duck population to which they had contact, but HPAI H5N8B viruses could not be detected despite cases in wild water birds and birds of prey around. According to our field observations, wild mallards seem to be the main duck species attracted by the sentinel mallards. In the light of the 2016–2017 HPAIV H5 epidemic in Europe, the mallard duck was one of the most frequently sampled bird species within the EU. However, the prevalence of HPAIV H5 within passive surveillance of mallards was lower than 5% in contrast to high H5N8B case fatalities in geese, swans and species of diving ducks (*Aythya*). In contrast, LPAIV was most frequently found in the mallard ducks and other, unspecified dabbling ducks (*Anas*) and rather rarely in swans and geese [28]. This data led to the main hypothesis that pre-exposure to AIV modulates lethal HPAIV H5N8 clade 2.3.4.4B infection and may persist undetected in dabbling duck populations.

The main aim of this experimental study was therefore to improve the current understanding of the role of dabbling ducks, particularly the mallard duck, in the dynamics of transmission of HPAIV H5N8 clade 2.3.4.4B. Besides, we wanted to evaluate the general suitability of seropositive mallard ducks as sentinel birds for the circulation of HPAIV in wild water bird populations. The number of naturally LPAIV-exposed and AI-seropositive ducks available for such experimental studies is extremely limited. It was therefore not possible to work with group sizes designed for hypothesis testing using regular biometrical planning. By contrast, the number of available animals dictated the biometrical design as an exploratory study with the purpose of generating hypotheses that need to be tested in further experiments or observational studies.

This experimental study showed that naturally LPAIV pre-exposed seropositive mallard ducks were susceptible towards HPAIV H5N8B infection although the majority of pre-exposed seropositive mallard ducks did not show any prominent clinical signs of infection except for diarrhea (shedding of green fecal matter) between 3 and 5 dpi (Table 2) and reduced activity. Green diarrhea and mild depression have also been described for Canada geese, pre-exposed to LPAIV and challenged with HPAI H5N1 virus [30]. However, one of the seropositive mallards died peracutely within 48 h without showing any clinical signs. Pathological and virological investigation proved that the HPAIV infection in this duck was systemic and most likely led to early liver failure (Table 2). We do not have any explanation why this duck developed a systemic infection by contrast to the other seropositive mallards. There was no indication for a secondary (bacterial) infection as judged by gross pathology. This duck was sampled positive for H1N3 on the 7 February 2018. In March and September the flock was infected with H5N3 and H4, respectively. At the date of sampling we did not detect viral genome in that duck, but it had a strong NP-antibody titre in September 2018. A similar phenomenon was reported when Baikal teals were infected with HPAI H5N8 virus A/Baikal teal/Kr/Donglim3/2014 [16]. Hepatic lipidosis (of the male mallard) of dietary origin is a very common finding in ducks, interpreted as background pathology and it was not checked whether it was caused by differential infectious, except influenzavirus, metabolic or toxic etiology.

LPAIV pre-exposed seropositive mallard ducks shed moderate viral loads of HPAIV H5N8B for a maximum of 7 days with a peak of shedding at 2–3 dpi. In contrast, the cloacal swabs of seropositive mallards only showed low viral loads for a shorter duration. This confirms outcomes of earlier studies with HPAIV H5N1 in pre-exposed mallard ducks [31,32] and also with heterosubtypic LPAI infection in pre-exposed mallards [33].

The viral loads shed by the seropositive mallards was sufficient to transmit virus to naïve contact ducklings. While 3 of the contact ducklings died within 4 days, one of them managed to clear the virus entirely and the humoral response was high against NP at 14 dpi.

The results of H5N8B infection of the seronegative Pekin ducks used as synspecific equivalents confirm and emphasize the general high “duck-virulence” of the virus strain as postulated earlier [12]. In contrast to the mild course of infection in the seropositive mallard ducks, case-fatality ratio of seronegative Pekin ducks was almost 100%. The difference in case-fatality ratio between adult seropositive mallards and adult seronegative Pekin ducks is highly noticeable. During the systemic infection, vRNA was detected in all tissues tested. The quill of wing feather proved to have a similar vRNA load as the intestines (Table 2). Immunohistochemistry confirmed the previously reported strong viral hepatotropism in most of the deceased ducks.

Survival of H5N8B challenge induced fully protective immunity against homologous H5N8B re-infection, at least when homologous infection occurred within a month. Reversion to a seronegative status, “seroreversion,” has previously been described for LPAIV for mallard ducks with a higher probability in juveniles [34]. Antibody responses in mature birds may be relatively long lasting, especially when the birds are continuously exposed to AIV antigens [34–37]. One week after the re-infection and 28 days after the first infection, the mallard ducks in our study became active in reproduction and 3 seropositive mallards even started to lay eggs. The yolks of these eggs, collected 34 days after the first infection, were also seropositive as confirmed by ELISAs (and no vRNA was detectable). Reportedly, maternal antibodies can persist in ducklings up to 4 weeks [38]. Therefore, the juvenile birds may be protected by homologous maternal-derived immunity against lethal HPAIV infection during their first weeks of life. Heterologous immunity, if induced by subsequent LPAIV infections and if cross-protective, may aid in clinical protection against HPAI infection thereafter.

Mallards have been described as a main wetland species with a high site fidelity that move depending on the availability of water [39] but are also capable of long-distance migration mainly in spring and fall with up to 549 km per day and a maximum distance of 1540 km [40]. It could be shown that LPAIV infection does not alter the behaviour of mallards [41]. It is difficult to estimate whether and if so how much the mild clinical symptoms in HPAIV-infected seropositive mallards impede their moving activity while shedding and therefore spreading virus. It cannot be excluded that such mallards may proceed during foraging trips into the perimeter of poultry holdings and visit ponds in urban areas [42]. Deposition of infectious HPAIV in those areas may increase the risk of exposure for poultry and humans. Viral contamination of the environment is a key element in AI transmission and spread [43,44]. In our experiments, infectious HPAIV H5N8B was also traceable in water and the stable environment until 6 dpi making infection by oral uptake a likely scenario although the virus is predominantly shed via the oropharynx and not the cloaca.

## Conclusion

Overall, the findings of our experimental study –a combination of a long-term field experiment with a laboratory *in-vivo* experiment – support earlier hypotheses of wild ducks of the *Anas* genus as asymptomatic virus carriers [1,31,32,45]. More specifically, LPAIV pre-exposed mallard ducks, which remain HPAIV H5-permissive may have the potential to play a role in HPAIV transmission and spread. However, virus excretion in surviving ducks that were re-infected with the same virus in a spatio-temporal correlation was unproductive and would not support a chain of virus transmission within a population. Despite pre-exposure to LPAIV, the mallard ducks were in general suitable as sentinel animals to pick up HPAIV circulating in wild water birds in the vicinity. Since virus shedding decreases rapidly within 7 days of infection, samples from such sentinel ducks should be taken as a combined oropharyngeal and cloacal swab or as a fecal swab in a weekly interval during high-risk periods.

## Supplementary Material

Supplemental Material
